# Inhibition of Monoacylglycerol Lipase Activity Decreases Glucose-Stimulated Insulin Secretion in INS-1 (832/13) Cells and Rat Islets

**DOI:** 10.1371/journal.pone.0149008

**Published:** 2016-02-11

**Authors:** Charles A. Berdan, Karel A. Erion, Nathan E. Burritt, Barbara E. Corkey, Jude T. Deeney

**Affiliations:** Department of Medicine, Obesity Research Center, Boston University School of Medicine, Boston, Massachusetts, United States of America; Monash University, AUSTRALIA

## Abstract

Lipid signals derived from lipolysis and membrane phospholipids play an important role in glucose-stimulated insulin secretion (GSIS), though the exact secondary signals remain unclear. Previous reports have documented a stimulatory role of exogenously added mono-acyl-glycerol (MAG) on insulin secretion from cultured β-cells and islets. In this report we have determined effects of increasing intracellular MAG in the β-cell by inhibiting mono-acyl-glycerol lipase (MGL) activity, which catalyzes the final step in triacylglycerol breakdown, namely the hydrolysis of MAG to glycerol and free fatty acid (FA). To determine the role of MGL in GSIS, we used three different pharmacological agents (JZL184, MJN110 and URB602). All three inhibited GSIS and depolarization-induced insulin secretion in INS-1 (832/13). JZL184 significantly inhibited both GSIS and depolarization-induced insulin secretion in rat islets. JZL184 significantly decreased lipolysis and increased both mono- and diacyglycerol species in INS-1 cells. Analysis of the kinetics of GSIS showed that inhibition was greater during the sustained phase of secretion. A similar pattern was observed in the response of Ca^2+^ to glucose and depolarization but to a lesser degree suggesting that altered Ca^2+^ handling alone could not explain the reduction in insulin secretion. In addition, a significant reduction in long chain-CoA (LC-CoA) was observed in INS-1 cells at both basal and stimulatory glucose following inhibition of MGL. Our data implicate an important role for MGL in insulin secretion.

## Introduction

Decades of research have demonstrated a critical role for insulin resistance in the manifestation of Type 2 Diabetes (T2D). However, a decline in β-cell function characterized by an inability to adequately respond to an acute glucose challenge, clearly distinguishes those who go on to develop for T2D from those who can maintain glucose homeostasis [1]. Our understanding of what causes β-cell failure is unclear, and may involve a multitude of factors including a decrease in β-cell mass, dedifferentiation of β-cells and apoptosis caused by exposure to excess nutrients [1]. Additionally, chronic exposure to elevated glucose and fatty acid (FA) has been shown to cause increased basal and reduced glucose-stimulated insulin secretion (GSIS), though the precise mediators of this effect remain unclear [2–5].

The consensus mechanism by which glucose stimulates insulin secretion involves a rise in intracellular free calcium ([Ca^2+^]_i_) that promotes the ‘triggering’ of insulin granule fusion with the plasma membrane via SNARE proteins [6]. Additional pathways and processes derived from glucose metabolism amplifies the insulin secretion induced by triggering [7]. Part of the amplification pathway of GSIS involves glycerolipid/free fatty acid (GL/FFA) cycling wherein glucose provides the glycerol backbone for FA esterification via α-glycerophosphate and also promotes lipolysis from lipid stores [8]. This GL/FFA cycle provides important signaling molecules, including long-chain (LC)-CoA, mono-acyl-glycerol (MAG) and di-acyl-glycerol (DAG) [8]. The targets of these lipid signals are diverse, including the stimulation of proteins involved in exocytosis [9–15], to broad kinases including PKC and ion channels [11].

While the role of DAG in GSIS has been studied extensively, the role of MAG has only recently garnered interest. We previously documented that acute treatment with mono-oleoyl-glycerol increases both basal and GSIS. Increased basal secretion depended on reactive oxygen species [16]. The Prentki group recently demonstrated the importance of the plasma membrane MAG lipase α/β-Hydrolase Domain-6 (ABHD6) as a regulator of GSIS [17]. Inhibition of this enzyme enhances insulin secretion at stimulatory glucose by increasing local MAG concentration and activating the exocytotic protein Munc-13a, which can bind both MAG and DAG. Mono-acyl-glycerol lipase (MGL), like ABHD6, is a serine hydrolase that removes the final FA during the breakdown of triacylglycerol to generate glycerol and FFA [18]. Research on this enzyme has focused on regulation of 2-arachidonoylglycerol synthesis in the brain, though it is known that the enzyme displays broad tissue distribution [19, 20]. Unlike ABHD6, MGL has been reported to be located in both plasma membrane and cytosol [18]. The contribution of this enzyme to GSIS has not been definitively established.

Herein, we demonstrate that pharmacological inhibition of MGL dose-dependently reduced glucose- and KCl-induced insulin secretion in both INS-1 cells and isolated rat islets. This reduction primarily occurred in the sustained, or second phase of GSIS. This coincided with a significant decrease in the LC-CoA pool and reduction in Ca^2+^ influx in response to both glucose and KCl.

## Materials and Methods

### Ethics Statement

This study was carried out in strict accordance with the recommendations in the guide for the care and use of Laboratory Animals and the National Institutes of Health. The protocol was approved by the Institutional Animal Care and Use Committee at Boston University Medical Center (Boston University Medical Center Animal Welfare Assurance: A-3316-01).

### Islets

Islets were isolated from male Sprague-Dawley rats (150–250 g) as previously described [5]. Briefly, the rat pancreas was excised and minced in 0.5 mL of Hank’s balanced salt solution (HBSS) containing 10 mM HEPES and adjusted to pH 7.4. The minced pancreas was then shaken in 5.5 mL HBSS with 3.3 mg collagenase P (1.7 units/mg) (Roche, Indianapolis, IN) in HBSS for 3 minutes at 37°C. The digested pancreas was then washed with HBSS supplemented with 0.1% defatted BSA (EMD Millipore, Billerica, MA) and further isolated by centrifugation through a Histopaque gradient (Sigma, St. Louis, MO). Isolated islets were cultured overnight in RPMI 1640 (11 mM glucose) without phenol red containing 10% FBS, 5 mM glutamine, 50 IU/mL penicillin, 50 μg/mL streptomycin. Islets were dissociated using accutase (Sigma, St. Louis, MO). Briefly, approximately 100 islets were incubated with 1 mL accutase at 37°C for 10 min and plated at a density of 2000–4000 cells/well in multiwell culture plates using matrigel (BD Biosciences, Billerica, MA).

### INS-1 (832/13) cells

INS-1 (832/13) cells were cultured in RPMI (11 mM glucose) with phenol red as above with the addition of 1 mM sodium pyruvate, 10 mM HEPES and 50 μM β-mercaptoethanol as previously described [5].

### Insulin Secretion

Insulin secretion was measured from INS-1 cells grown for at least 3 days to approximately 85% confluence in 48- and 96-well plates as we have described in previous work [16]. Briefly, INS-1 cells were preincubated with RPMI containing 2 mM glucose without FBS for 2 hours prior to a 30-minute preincubation with 2 mM glucose in Krebs-Ringer bicarbonate buffer (KRB) at 37°C. The KRB buffer contained 119 mM NaCl, 4.6 mM KCl, 5 mM NaHCO_3_, 2 mM CaCl_2,_ 1 mM MgSO_4_, 0.15 mM Na_2_HPO_4_, 0.4 mM KH_2_PO_4_, 20 mM HEPES, 0.05% BSA, pH 7.4. Dissociated islets and whole rat islets (6 islets/tube) were preincubated in 3 mM glucose in KRB at 37°C for 30 minutes prior to measuring secretion. Following preincubations, INS-1 cells and islets were cooled on ice before experimental solutions were added. Cells were incubated for 60–120 min at 37°C, cooled, and sampled for insulin secretion. Insulin was measured by HTRF insulin assay (CisBio, Bedford, MA). Islet perifusions were performed as described previously [21].

### MGL inhibitor preparations

JZL184 (Cayman Chemical, Ann Arbor, MI), MJN110 (Sigma, St. Louis, MO) and URB602 (Cayman Chemical, Ann Arbor, MI) were prepared as a DMSO stocks. For experiments stocks were diluted into KRB or RPMI at 37°C while vortexing. The final concentration of DMSO was 0.1% or less.

### Neutral Lipid Quantification

INS-1 cells were grown to 1.2 million cells/well in 12-well plates. 48 hours before extraction, cells were cultured in RPMI (1 mL/well) containing 5 μCi/mL uniformly labeled C^14^-glucose. Prior to extraction radio-labeled cells were incubated as for insulin secretion. Cells were extracted with 3 mL chloroform methanol (2:1) and 0.75 mL 168 mM NaCl 0.9 mM HCl. The organic layer was collected and dried under air and separated using single dimension TLC on silica gel G plates using hexane:ethyl ether:acetic acid (70:30:1; v/v) as the development system. Individual lipid species were located by autoradiography and co-migration with authentic standards. Radioactive bands were scraped and radioactivity was quantified using liquid scintillation spectrometry. Parallel unlabeled wells were used for normalization by cell count.

### Glycerol release

Glycerol was measured enzymatically using glycerokinase and α-glycerophosphate dehydrogenase from the same final incubation solutions removed from INS-1 cells to monitor JZL184-induced insulin release described above. NADH produced in the assay was detected by luminescence as previously described [22].

### Ca^2+^ Determination

Intracellular Ca^2+^ was measured in INS-1 cells and rat islets using fura-2 AM (Invitrogen, Carlsbad, CA). INS-1 cells were seeded in 35 mm glass bottom dishes (MatTek, Ashland, MA) and cultured as previously described to a density of approximately 200,000 cells/well. Cells were then rested in 2 mM glucose RPMI without FBS for 2 hours followed by a 30-minute incubation in 2 mM glucose KRB with 2 μM fura-2 AM and 0.1% pluronic acid (Thermo Fisher Scientific, Waltham, MA). Whole islets were loaded with 2 μM fura-2 AM while shaking at 37°C in 3 mM glucose KRB for 30 minutes. After loading both cells and islets were incubated for 15 minutes in KRB without fura-2 AM to complete cleavage of the loaded fura-2 AM ester prior to mounting onto the stage of an Olympus DSU spinning disk microscope. A stage chamber maintained a 37°C temperature throughout the experiment. Fluorescence images were obtained at 20X magnification every 10 seconds with excitation wavelengths of 340 and 380 nm and dual emission of 510 nm.

Whole islets were perifused using a high precision multichannel pump (Ismatec, Wertheim, Germany) at a flow rate of 1 mL/min to introduce test solutions. Measurements were quantified using ImageJ. For INS-1 cells, test solutions were introduced via pipette with concentrated solutions. 16–32 individual INS-1 cells per experiments were analyzed using NIS Elements software. Because baseline conditions were identical prior to addition of test solutions, all baselines were averaged and responses were normalized.

### LC-CoA measurement

INS-1 cells were grown to 1.2 million cells/well in 12-well plates and incubated as for insulin release. Cells were exposed to 10 μM JZL184 for up to 60 minutes and media sampled for insulin release. Cells were then quick frozen in liquid nitrogen and stored at -80°C until processed for LC-CoA analysis. Frozen cells were thawed on ice in 1% trichloroacetic acid (TCA) containing 3.75 mM DTT. Cells were centrifuged (12,000x g, 3 minutes) and washed 3 times with equal volumes of ether to remove TCA from the precipitate containing LC-CoA. The precipitates were hydrolyzed in 3.75 mM K_2_HPO_4_ buffer pH 11.5 at 55°C for 10 min to liberate free CoA from the LC-CoA. Free CoA was then measured enzymatically using the α-ketoglutarate dehydrogenase reaction as described previously [23]. Standard curves were produced from varying concentrations of both ether washed free CoA and hydrolyzed LC-CoA in order to account for recovery during sample processing. NADH produced in the assay was then detected by luminescence using NADH-dependent bacterial luciferase as detailed by Peyot et al [22].

### Statistical analysis

Data represent the mean ± standard error. Two-way analysis of variance (ANOVA) on log-transformed data was used to assess the main effects of glucose (low vs. high) and dose (or time as appropriate) on insulin secretion, with repeated measured on the latter (GraphPad Prism 5). When the main effect of dose (or time) or interaction between glucose and drug/time on insulin was significant (*p* < 0.05), the effect of drug/time within each glucose concentration was tested by a one-way repeated measures ANOVA followed by post-hoc Dunnett’s tests to compare each value to the control group. When indicated, paired student’s t-tests were used to determine significance of glucose on control. Values of *P* < 0.05 were considered statistically significant.

## Results

### Inhibition of MGL reduced GSIS

**Fig 1:**
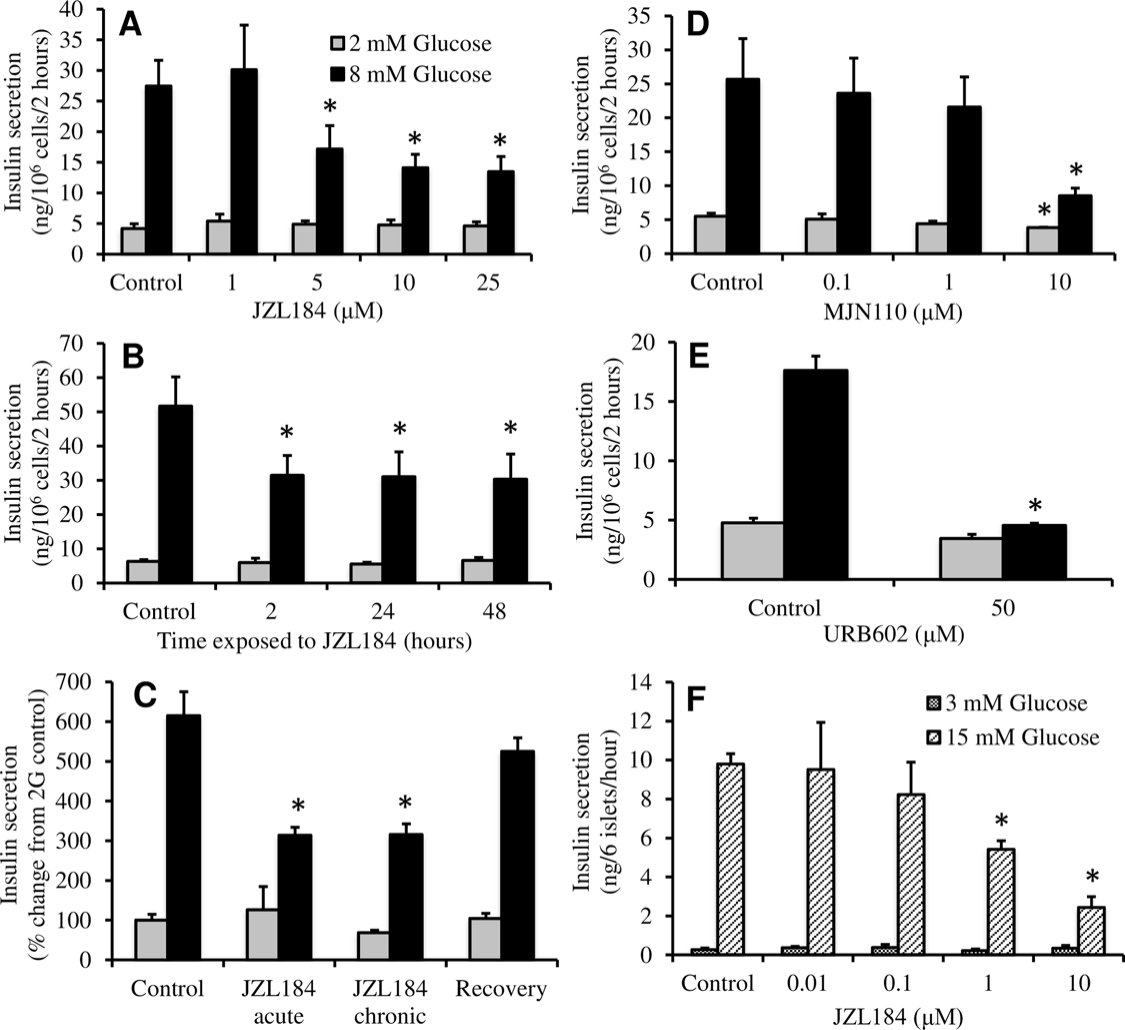
Inhibition of MGL reduced GSIS in INS-1 cells and isolated rat islets. (A) JZL184 dose-dependently reduced GSIS during static incubations in INS-1 cells (n = 3). (B) JZL184 (10 μM) reduced GSIS in INS-1 cells regardless of the temporal exposure (n = 3). (C) INS-1 cells demonstrated a partial recovery of GSIS following the removal of JZL184 (10 μM) for 2 hours (n = 3). (D) MJN110 dose-dependently reduced GSIS during static incubations in INS-1 cells (n = 4). (E) URB602 (50 μM) inhibited GSIS during static incubation in INS-1 cells (n = 3). (F) JZL184 dose-dependently reduced GSIS in isolated whole rat islets during a static incubation (n = 3). Error bars represent S.E. from independent experiments (*, *p*<0.05).

The effect of pharmacological inhibition of MGL on GSIS was determined using JZL184, an MGL inhibitor that acts via carbamoylation of a serine nucleophile [24]. Acute stimulation of INS-1 cells with glucose increased insulin secretion by approximately 5-fold over a two-hour static incubation period. A marked inhibition was seen at 5 μM and maximal inhibition was observed at 10 μM (Fig 1A). JZL184 did not significantly affect basal insulin secretion at any concentration tested. Chronic exposure of INS-1 cells to JZL184 did not result in any further inhibition of GSIS, and also ensured that the decrease was not due to toxicity (Fig 1B). Removal of JZL184 for 2 hours resulted in a 60% recovery of GSIS, indicating at least partial reversibility (Fig 1C).

Since JZL184 has also been shown to inhibit fatty acid amide hydrolase (FAAH), albeit at much higher concentrations, we used another inhibitor of MGL, MJN110, which reportedly does not inhibit FAAH [24, 25]. Acute exposure of INS-1 cells to 10 μM MJN110 resulted in a similar reduction in GSIS compared to JZL184 (Fig 1D). Finally, we observed a reduction in GSIS over a two-hour period using 50 μM URB602, a third MGL inhibitor (Fig 1E) [26].

**Fig 2:**
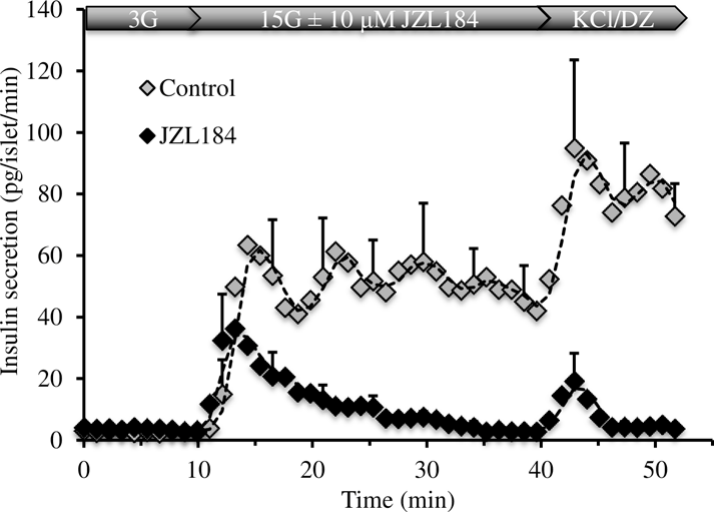
JZL184 reduced sustained insulin secretion during glucose stimulation in whole isolated rat islets. Islets were perifused with basal glucose (3 mM) KRB buffer for approximately 10 minutes followed by a 30-minute perifusion with 15 mM glucose with or without 10 μM JZL184. At 40 minutes the islets were perifused with 15 mM glucose KRB with 0.4 mM DZ and 30 mM KCl with or without 10 μM JZL184. n = 3 independent experiments from different islet isolations. Error bars represent S.E. and are displayed every 4^th^ data point for clarity. All points after 23.1 minutes were significantly different (*p*<0.05).

While INS-1 cells retain many of the characteristics of normal β-cells, the fact that they are a tumor cell line necessitates confirmation in primary β-cells. Isolated rat islets showed an approximate 35-fold increase in insulin secretion when stimulated with 15 mM glucose (Fig 1F). JZL184 at 1 and 10 μM caused an approximately 50 and 75% reduction of GSIS, respectively. Perifusion of islets in which the glucose concentration was raised from 3 to 15 mM with and without 10 μM JZL184 revealed that inhibition of insulin secretion by JZL184 was greater during the sustained phase of GSIS (Fig 2). Perifusion with a high concentration of KCl, which depolarizes the plasma membrane and increases [Ca^2+^]_i_, resulted in a sustained increase in control islets while only a transient spike was observed in the islets exposed to JZL184.

### JZL184 inhibits depolarization-induced insulin secretion

**Fig 3:**
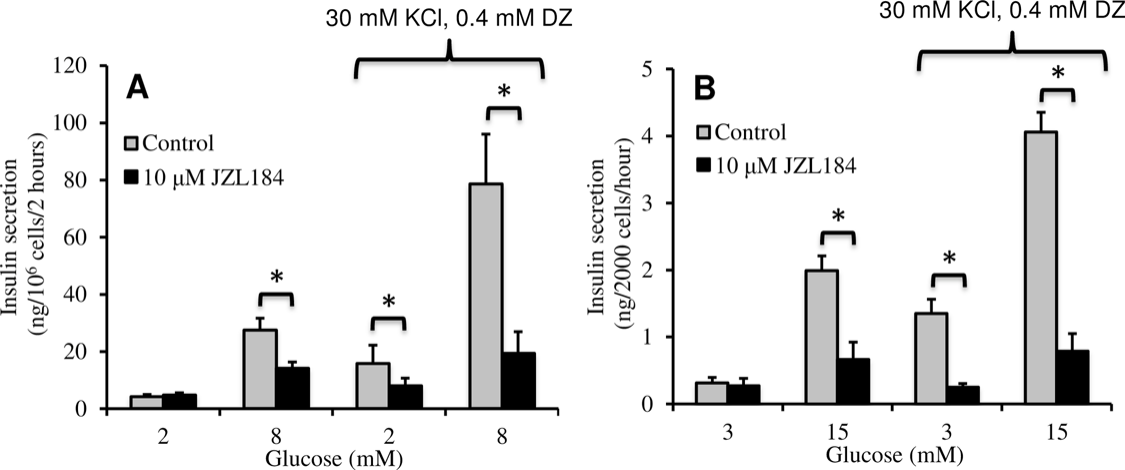
JZL184 inhibited KCl-induced insulin secretion. Insulin secretion from batch incubations of (A) INS-1 cells and (B) dissociated rat islets (n = 3 independent experiments for both A and B). Error bars represent S.E. (*, *p*<0.05).

In order to test whether direct depolarization of the plasma membrane could recover the defect in GSIS observed with MGL inhibitors, we used a high concentration of KCl plus the ATP-sensitive K^+^ (K_ATP_)-channel agonist diazoxide (DZ). Insulin secretion is increased under these conditions with or without stimulatory glucose via a sharp peak and mildly elevated sustained phase of cytosolic [Ca^2+^]. Additionally, it is known that glucose can stimulate insulin secretion in a concentration dependent manner under these conditions [27]. We found that JZL184 completely inhibited KCl/DZ-induced insulin secretion in INS-1 cells at both basal and stimulatory glucose (Fig 3A). The MGL inhibitors MJN110 and URB602 also inhibited KCl-induced insulin secretion (data not shown). Additionally, pharmacological inhibition of MGL completely blocked depolarization-induced insulin secretion in dissociated islet cells (Fig 3B).

### JZL184 reduced glycerol release and increased MAGs and DAGs in INS-1 cells

**Fig 4:**
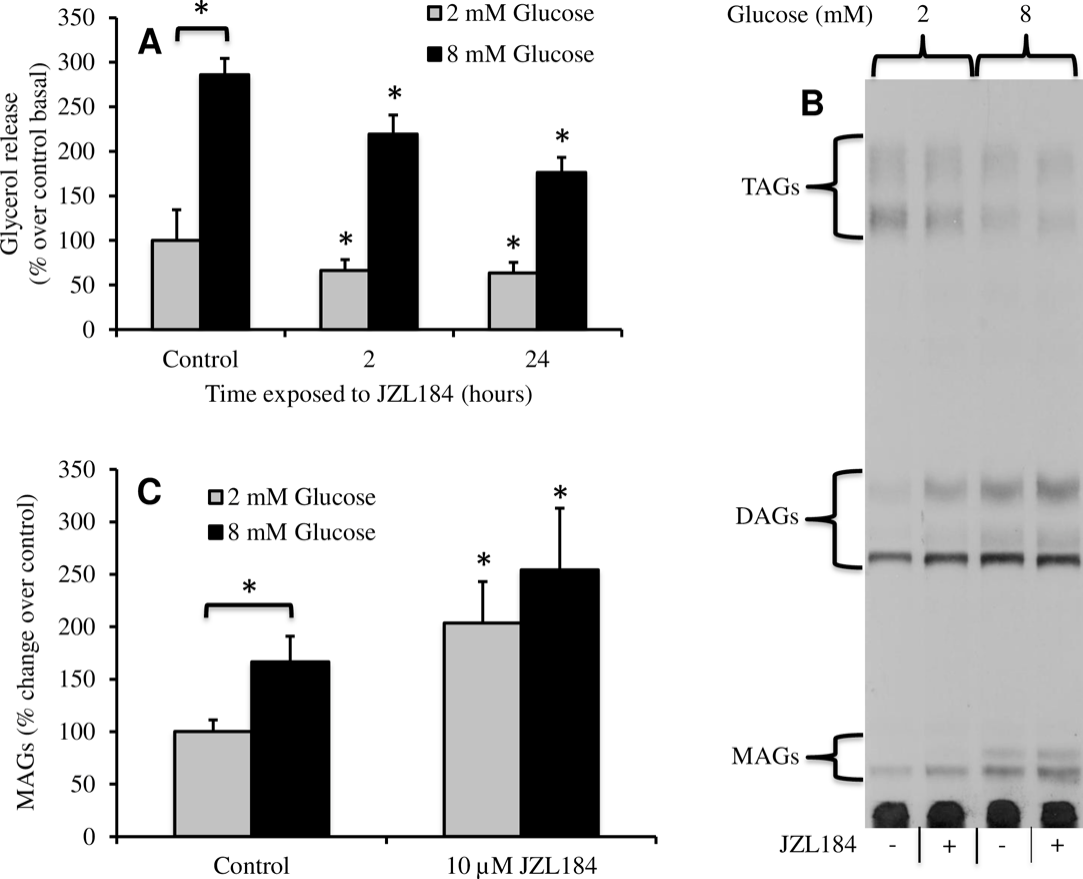
Exposure to JZL184 reduced glycerol release and increased MAG species in INS-1 cells. (A) Incubation with 10 μM JZL184 time-dependently reduced glycerol release at both basal and stimulatory glucose (n = 6). (B) Acute exposure to JZL184 for 30 minutes increased total MAG species at both basal and stimulatory glucose. (C) Quantitation of total MAGs from 4 experiments. Error bars represent S.E. from independent experiments (*, *p*<0.05).

Glucose stimulates lipolysis from intracellular lipid stores in β-cells [28]. The sequential hydrolysis of fatty acids from triacylglycerol led to increases in MAGs and DAGs. Hydrolysis of MAG generates glycerol, which is released from the cells, and a FFA, which can be released or re-esterified [8]. Blocking MGL activity with JZL184 should decrease glycerol release from the cell following stimulation of lipolysis with glucose. Acute stimulation with glucose caused a three-fold increase in release of glycerol from INS-1 cells during a 2 hour incubation period (Fig 4A). Inhibition of MGL with JZL184 reduced glycerol release by 20% at both basal and stimulatory glucose. A 24-hour exposure resulted in a more potent inhibition of approximately 50%.

To further support this concept, we used thin layer chromatography to determine the affect of JZL184 on lipid species at both basal and stimulatory glucose. [1-^14^C]-Glucose was incubated with INS-1 cells overnight to label the lipid pool. Stimulation with 8 mM glucose for 30 min resulted in a 50% increase in total MAGs (Fig 4B and 4C), consistent with a previous report [29]. At basal glucose, JZL184 nearly doubled total MAG species. At stimulatory glucose, JZL184 induced a greater increase than that seen with glucose alone. DAG species also appeared to increase when cells were exposed to JZL184, regardless of the glucose concentration, suggesting a block in GL/FFA cycling.

### Alterations in [Ca^2+^]_i_ with JZL184 in response to glucose and KCl/DZ

**Fig 5:**
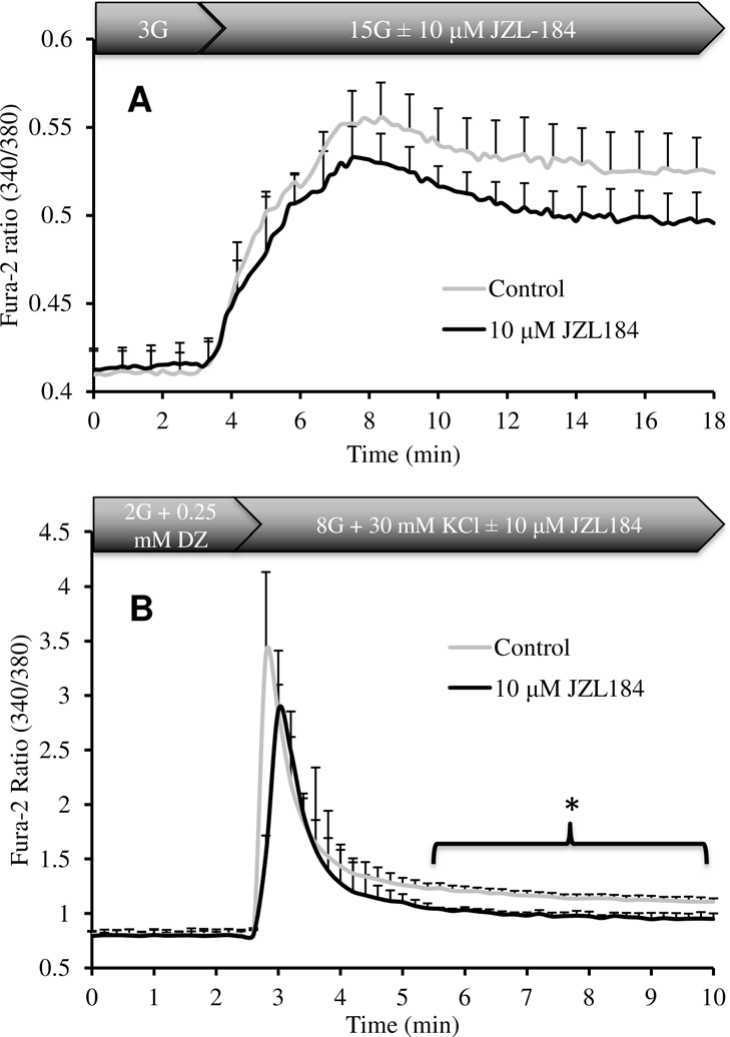
Small decreases in Ca^2+^ influx following exposure to JZL184 in isolated rat islets and INS-1 cells. (A.) Islets were brought from 3 mM glucose to 15 mM glucose with or without 10 μM JZL184 and cytosolic Ca^2+^ was documented using the probe fura-2 (n = 4). (B) INS-1 cells were brought from 2 mM glucose plus 0.25 mM DZ to 8 mM glucose with 0.25 mM DZ and 30 mM KCl with or without 10 μM JZL184 (n = 3). Ca^2+^ levels are expressed as the fura-2 ratio at 340/380 nm or the increment above basal in response to treatment (peak and sustained).

The final common step in the triggering pathway of GSIS and depolarization is a rise in cytosolic [Ca^2+^]_i_, which directly promotes the fusion of insulin granules with the plasma membrane [30, 31]. The ability of JZL184 to inhibit both GSIS and KCl/DZ induced insulin secretion implied a strong likelihood that [Ca^2+^]_i_ was reduced in response to these stimuli. We tested this concept using the cytosolic [Ca^2+^]_i_ indicator fura-2. Rat islets were perifused with basal glucose followed by an increase to 15 mM with and without JZL184, similar to what was done in the insulin secretion perifusions. A clear sustained rise in cytosolic [Ca^2+^]_i_ was observed in response to 15 mM glucose (Fig 5A). However, exposure of the islets to glucose plus JZL184 significantly inhibited this increase over the time course observed. Similarly, the peak increase in intracellular [Ca^2+^]_i_ induced by KCl/DZ was slightly inhibited by JZL184 and the sustained phase was decreased by more than half in INS-1 cells (Fig 5B).

### Decreased LC-CoA in cells with reduced MGL activity

**Fig 6:**
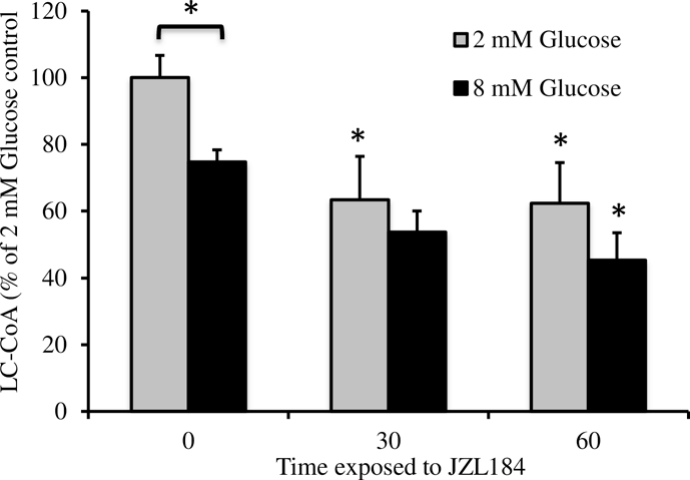
Significant reduction in total LC-CoA following acute treatment of INS-1 cells with JZL184. Cells were exposed to JZL184 at both basal and stimulatory glucose and LC-CoA was measured as described in the methods (n = 3). Error bars represent S.E. from independent experiments (*, *p*<0.05).

We have previously documented a key role of LC-CoA as a metabolic coupling factor in GSIS [11, 32]. An increase in glucose flux in the β-cell stimulates an elevation of TCA cycle intermediates and the conversion of citrate to malonyl-CoA in the cytosol [33]. The inhibition of CPT-1 by malonyl-CoA results in an increase in cytosolic LC-CoA, which may be rapidly converted to other downstream fatty acid signaling molecules including phosphatidic acid, DAG and triglycerides. Increased glucose flux also stimulates lipolysis and GL/FFA cycling, which both produces and consumes LC-CoA [28]. The net result is a marked reduction of the major mitochondrial pool of LC-CoA due to malonyl-CoA inhibition of LC-CoA entry, and a transient rise in cytosolic levels. Acute stimulation of INS-1 cells with 8 mM glucose caused a 25% reduction in total LC-CoA levels, likely a result of a reduction in the mitochondrial pool (Fig 6). Incubation with JZL184 for 30 minutes caused a further reduction of LC-CoA at both basal and stimulatory glucose of approximately 40% and 25% respective to their own controls. A 60-minute incubation in JZL184 induced no further reduction compared to 30 minutes.

## Discussion

Herein we have demonstrated a role for MGL activity in GSIS. Inhibition of MGL resulted in a pronounced defect in the sustained phase of GSIS. Importantly, we were able to demonstrate that this effect was partially a result of reduced [Ca^2+^]_i_ in response to glucose. Consistent with this, depolarization of the plasma membrane with KCl/DZ resulted in a similar but slightly smaller peak rise in [Ca^2+^]_i_ with concurrent JZL184 incubation and decreased the sustained phase [Ca^2+^]_i_ rise. Inhibition of MGL also caused a reduction in LC-CoA, an important coupling factor derived endogenously from lipid stores during lipolysis._._

The role of LC-CoA in GSIS has been debated in the literature [32]. The well documented increase in malonyl-CoA competitively inhibits CPT-1, effectively blocking the transfer of long-chain fatty acids into the mitochondria for β-oxidation [34]. The resulting increase in cytosolic LC-CoA is then directed into complex lipid signaling molecules such as DAG and phosphatidic acid and also consistent with increased triglyceride stores for which LC-CoA is a precursor. In addition, LC-CoA itself can directly induce exocytosis and serves as a substrate for the acylation of proteins [11]. The reduction of LC-CoA we observed with glucose is consistent with the fact that the CoA pool is largest in the mitochondria, thus inhibition of CPT-1 causes a significant reduction in total LC-CoA even though cytosolic LC-CoA is likely elevated, at least transiently. The reduction in LC-CoA caused by inhibition of MGL likely plays a significant role in the documented reduction in insulin secretion due to a late direct or acylation effect on exocytosis. Interestingly, inhibition of MGL potently reduced the second phase of GSIS, which may be dependent on granule movement to the plasma membrane, whereas the initial phase of secretion that was less affected reflects largely already docked granules [6].

The components that are responsible for sustained secretion are not entirely clear, although complex lipid signaling species play an important role. On the other hand the triggering pathway, which is primarily thought to be responsible for the first phase of secretion, is mainly driven by [Ca^2+^]_i_ influx. Our data suggest that the triggering of insulin secretion requires signals in addition to [Ca^2+^]_i_, which we have previously suggested [5]. It is unclear from our data whether the effect of MGL inhibitors was due to a missing lipid signal or because of the buildup of an inhibitory signaling molecule. The most likely candidate for signal buildup is one or several MAG species, though we cannot rule out the possibility that a DAG species, which are also elevated as a result of the MGL inhibitor, may also play a role. It should be noted that McGarry demonstrated that there was no GSIS in fasted perfused pancreas without addition of FFA [35].

Three different MGL inhibitors significantly decreased both GSIS and depolarization-induced secretion. Detailed studies of one inhibitor, JZL184 indicated that it impacted signaling at both LC-CoA and cytosolic Ca^2+^. There is a potential relationship between these two entities as LC-CoA has been shown to affect K_ATP_ channels and L-type Ca^2+^ channels. Multiple groups have demonstrated that LC-CoA activates K_ATP_ channel and blunts Ca^2+^ entry [36, 37]. However, our data documents a decrease in LC-CoA and a decrease in [Ca^2+^]_i_, which goes in the opposite direction and suggests that the MGL inhibitors are not affecting K_ATP_ channels. Additionally, because an affect is still observed with KCl and diazoxide, which takes offline the K_ATP_ channel, an affect on the K_ATP_ channel is unlikely. A role for LC-CoA in mediating Ca^2+^ influx through L-type Ca^2+^ channels has been documented [38]. However, we cannot rule out the possibility that MGL inhibitors affect [Ca^2+^]_i_ via an affect on Ca^2+^ stores. These data differed from other published studies showing that ABHD6 is the primary monoglyceride lipase in β-cells and is essential for GSIS [17].

Recent publications by the Prentki group may clarify these apparent discrepancies. They observed differences in cellular distribution and in particular that ABHD6 is localized to the plasma membrane whereas MGL is not. Most importantly, they recently documented major non-specificity of different lipase inhibitors [39]. First, JZL184 was found to inhibit not only MGL, but also two other MG lipases, fatty acid amide hydrolase (FAAH) and carboxyesterase-1 (CES1) and to a lesser extent ABHD6. Thus our data do not identify a single target for inhibitor activity, although it is clear from the increase in MAGs following treatment that lipase activity was effectively inhibited. Secondly, the Prentki group compared enzymes and inhibitors in mouse and human cells. Many differences in inhibitor effectiveness were observed between species. Since we used rat, the possibility of additional differences must be considered. Finally, our previous studies with exogenously added mono-oleoyl-glycerol showed an increase in basal secretion that we did not find in response to intracellular accumulation of MAGs suggesting either an effect of the specific species or specific localization of the internally generated MAGs [16]. It is interesting to note that exogenous addition of mono-oleoyl-glycerol increased LC-CoA, whereas inhibition of MGL decreased LC-CoA and did not affect basal secretion. This strongly implicates LC-CoA as an important regulator of basal insulin secretion and builds upon numerous studies showing that chronic incubation of β-cells with exogenous lipid increases basal insulin secretion [4].

Several previous papers have characterized the role of MAGs and MGL in the regulation of GSIS. Li et al. used the MGL inhibitor URB602 and observed an enhancement of insulin secretion at both basal and stimulatory glucose concentrations in MIN6 cells and human islets [40]. This increase in insulin secretion coincided with a rapid increase in [Ca^2+^]_i_ influx, which is not sustained. In contrast, we observed a marked inhibition of both glucose and KCl-induced insulin secretion using the same inhibitor, URB602. Several factors may explain the apparent discrepancy between this result and our own data. Our experiments were conducted over a longer time frame and therefore a transient increase in insulin secretion may have been masked by a later prolonged decrease, which Li et al. also observed. While we cannot conclude that the pharmacological inhibitors used in our study were specific, we were able to measure both a reduction in glycerol secretion and a definitive increase in total MAG accumulation using thin layer chromatography, thereby demonstrating that indeed the drugs used did inhibit MAG hydrolysis, as expected.

A recent report by Zhao et al. clearly demonstrates that pharmacological or genetic inhibition of the plasma membrane hydrolase, ABHD6, stimulates GSIS possibly by action of increased MAGs binding to the exocytotic protein Munc13-1 [17]. While it was argued that ABHD6 is the predominant lipase that breaks down MAG in β-cells and that MGL is expressed at low levels, others have demonstrated that MGL is expressed in both human and rodent β-cells [40]. These authors also demonstrate that in INS-1 cells, 1 μM JZL184 increases MAG species, but has no effect on insulin secretion, similar to what we have shown (Fig 1A). Thus INS-1 cells require a higher concentration than islets, possibly due to their large intracellular lipid stores when cultured at high glucose [5].

In summary, we have demonstrated that pharmacological inhibition of MGL reduces both KCl and glucose induced insulin secretion. This reduction is the result of processes that may partially involve the classical Ca^2+^-dependent pathway via which glucose and KCl induce insulin secretion as well as a failure to increase cytosolic LC-CoA. We cannot rule out the possibility that the build-up of specific MAG species in a specific location may be responsible for the observed inhibition of GSIS. Alternatively, lack of specificity of the lipase inhibitors may alter other lipid species not measured. Further research will be needed to determine the role of specific MAG species on GSIS.
